# Advances in Substrate Recognition, Catalytic Mechanisms, and Engineering Applications of Alginate Lyases

**DOI:** 10.3390/polym18202523

**Published:** 2026-10-15

**Authors:** Zhen Li, Xingyu Liu, Wenjie Tang, Kangsheng Xie, Rui Wang, Peng Lei, Yian Gu, Hong Xu, Liang Sun

**Affiliations:** College of Food Science and Light Industry, Nanjing Tech University, Nanjing 211816, China; lzzzzz@njtech.edu.cn (Z.L.); lxy2001@njtech.edu.cn (X.L.); 202421057024@njtech.edu.cn (W.T.); 202421126033@njtech.edu.cn (K.X.); ruiwang2013@njtech.edu.cn (R.W.); lei-peng@njtech.edu.cn (P.L.); yian.gu@hotmail.com (Y.G.); xuh@njtech.edu.cn (H.X.)

**Keywords:** alginate lyase, alginate oligosaccharides, protein engineering, antibiofilm therapy

## Abstract

Alginate lyases depolymerize alginate via β-elimination and serve dual roles in brown-algal biorefining and antibiofilm therapy. Brown-algal and bacterial alginates differ substantially in O-acetylation, yet this key structural distinction and its implications for enzyme performance remain underappreciated. This review systematically integrates the complete causal chain from alginate polymer structure through enzyme recognition, catalytic mechanism, and protein engineering to bidirectional applications. We cover PL family classification, the structural basis of substrate specificity, engineering strategies including two-step activity stability optimization and AI-assisted design, and recent progress in tailored alginate oligosaccharide preparation and cystic fibrosis treatment. Our central analysis identifies a widespread substrate mismatch, as most enzymological characterization relies on non-acetylated sodium alginate, whereas the clinical target is heavily acetylated bacterial alginate. This review establishes acetylated substrate adaptation as the critical gap between fundamental enzymology and medical translation and outlines future directions including multi-objective optimization and AI-driven enzyme design.

## 1. Introduction

Biofilms are structured microbial communities attached to biotic or abiotic surfaces and embedded in a self-produced extracellular polymeric substance (EPS) matrix. Their tolerance to antibiotics and host immune defenses is a major cause of refractory chronic infections [1]. Within biofilm EPS, extracellular polysaccharides are considered key determinants of biofilm architecture and barrier function, due to their high viscoelasticity, water retention, and ion-exchange capacity [2]. *Pseudomonas aeruginosa* (PA) is a leading cause of hospital-acquired infections and chronic lung infections in cystic fibrosis (CF) patients. In the CF lung, PA undergoes mucoid conversion and secretes large quantities of alginate-type EPS, which forms a physical barrier against antibiotic penetration and immune clearance and is strongly associated with irreversible lung function decline [3].

From a polymer science perspective, alginate is a distinctive bio-based anionic polysaccharide. Its linear backbone consists of two epimers, β-D-mannuronate (M) and α-L-guluronate (G), arranged non-periodically. The block composition of MM, GG, and MG sequences, together with the molecular weight and O-acetylation pattern, governs the gelling, rheological, and biological properties of alginate. Owing to its excellent biocompatibility, mild divalent-cation-mediated gelation, and tunable degradation, alginate is widely used in wound dressings, drug delivery, cell encapsulation, and tissue engineering [4].

Alginate lyases (EC 4.2.2.3) cleave the glycosidic bonds of alginate through β-elimination, yielding oligosaccharide products that terminate in 4-deoxy-L-erythro-hex-4-enopyranuronosyluronate, an unsaturated uronate. Compared with chemical acid hydrolysis, enzymatic depolymerization proceeds under mild conditions without strong acids or bases, gives products with well-defined configurations, and allows the degree of polymerization (DP) of the products to be directed by selecting enzymes with different substrate specificities [5,6]. Since their first description in the 1960s, studies of alginate lyases have advanced from phenomenological enzymology to the dissection of molecular mechanisms and, most recently, to rational protein design. Since 2000, the CAZy database’s systematic classification framework, based on amino acid sequence similarity and fold type, combined with high-resolution crystal structures of representative enzymes from multiple families, has substantially advanced understanding of the catalytic mechanisms and substrate-recognition rules of this enzyme family [7,8].

In parallel, the application landscape of alginate lyases has kept expanding. On the industrial side, alginate lyases are the key bottleneck for converting brown-algal biomass into value-added products such as alginate oligosaccharides (AOS), 4-deoxy-L-erythro-5-hexoseulose uronate (DEH), and bioethanol. Wargacki et al. [9] transplanted the 36 kb alginate-metabolism gene cluster of *Vibrio splendidus* into *Escherichia coli* to build a synthetic microbial platform that ferments brown algae directly into ethanol, and Enquist-Newman et al. [10] subsequently achieved full utilization of brown-algal sugars in *Saccharomyces cerevisiae*. On the medical side, Alkawash et al. [11] demonstrated as early as 2006 that exogenous alginate lyase markedly enhances antibiotic killing of mucoid PA biofilms, thereby opening the antivirulence paradigm of enzyme-aided antimicrobial therapy. In the two decades since, new lyase families have kept emerging, structures and mechanisms have become increasingly clear, and engineering strategies have iterated rapidly; however, reviews that systematically integrate the complete causal chain, extending from polymer structure and enzyme-level recognition through catalytic mechanism and engineering strategy to bidirectional applications, remain scarce.

**Figure 1:**
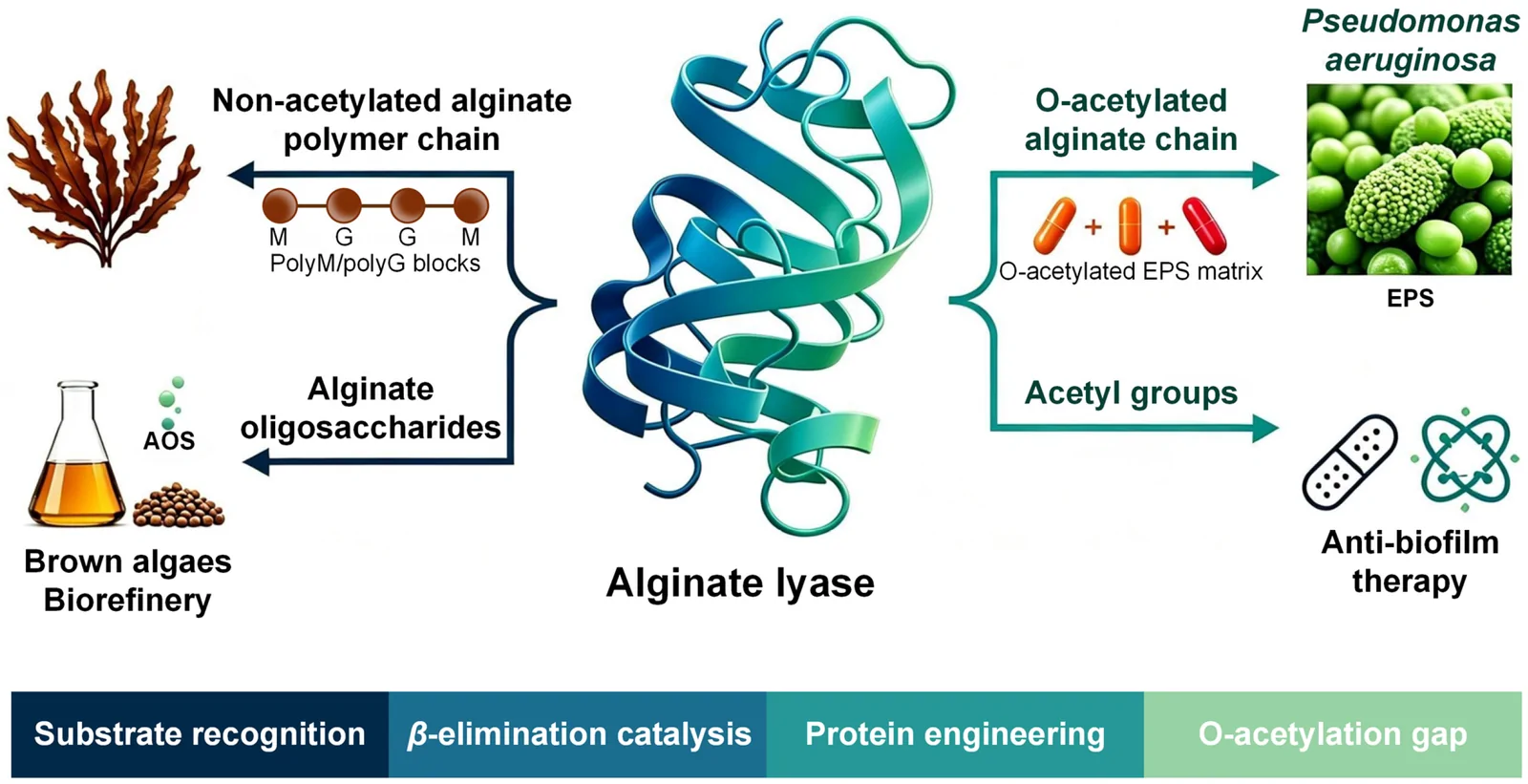
Schematic overview of alginate lyase as a single enzyme connecting dual substrate sources and bidirectional applications. Brown-algal alginate is non-acetylated with variable M/G block composition, whereas bacterial *Pseudomonas aeruginosa* alginate is heavily O-acetylated at O-2 and O-3 positions and predominantly polyM-rich.

At the molecular level, these two application branches rest on a shared foundation. A single catalytic architecture operates throughout, comprising a β-elimination center assembled from a small set of family-specific residues and a linear array of substrate-binding subsites numbered −n to +n. The substrate is likewise a single polysaccharide whose properties vary only in O-acetylation status and in the M/G block pattern imposed by its biological origin. Biorefining leverages this relationship to convert a polymer of defined block structure into a controlled product distribution, whereas antibiofilm therapy applies the same catalytic logic to a matrix whose acetylation and block composition differ substantially from those of the industrial substrate. Against this background, this review adopts a unified perspective centered on a single enzyme class that connects two substrate sources and serves two application directions. Starting from alginate polymer structure, it follows a logical progression from substrate recognition and catalytic mechanism through protein engineering to biorefining and antibiofilm applications (Figure 1). The fundamental difference in O-acetylation between bacterial and brown-algal alginate is the central issue linking enzymology, industrial use, and clinical translation, and it represents the key gap that this review aims to address. The review concludes by discussing the opportunities and challenges facing the frontier directions of artificial-intelligence-driven enzyme design, dispersed-cell safety, enzyme immobilization, and continuous production, with the aim of providing a systematic reference for research on polymer-degrading enzymology, marine biorefining, and anti-infective therapy.

## 2. Structure, Sources, and Biological Functions of Alginate

### 2.1. Chemical Structure and the Block-Copolymer Mode

**Figure 2:**
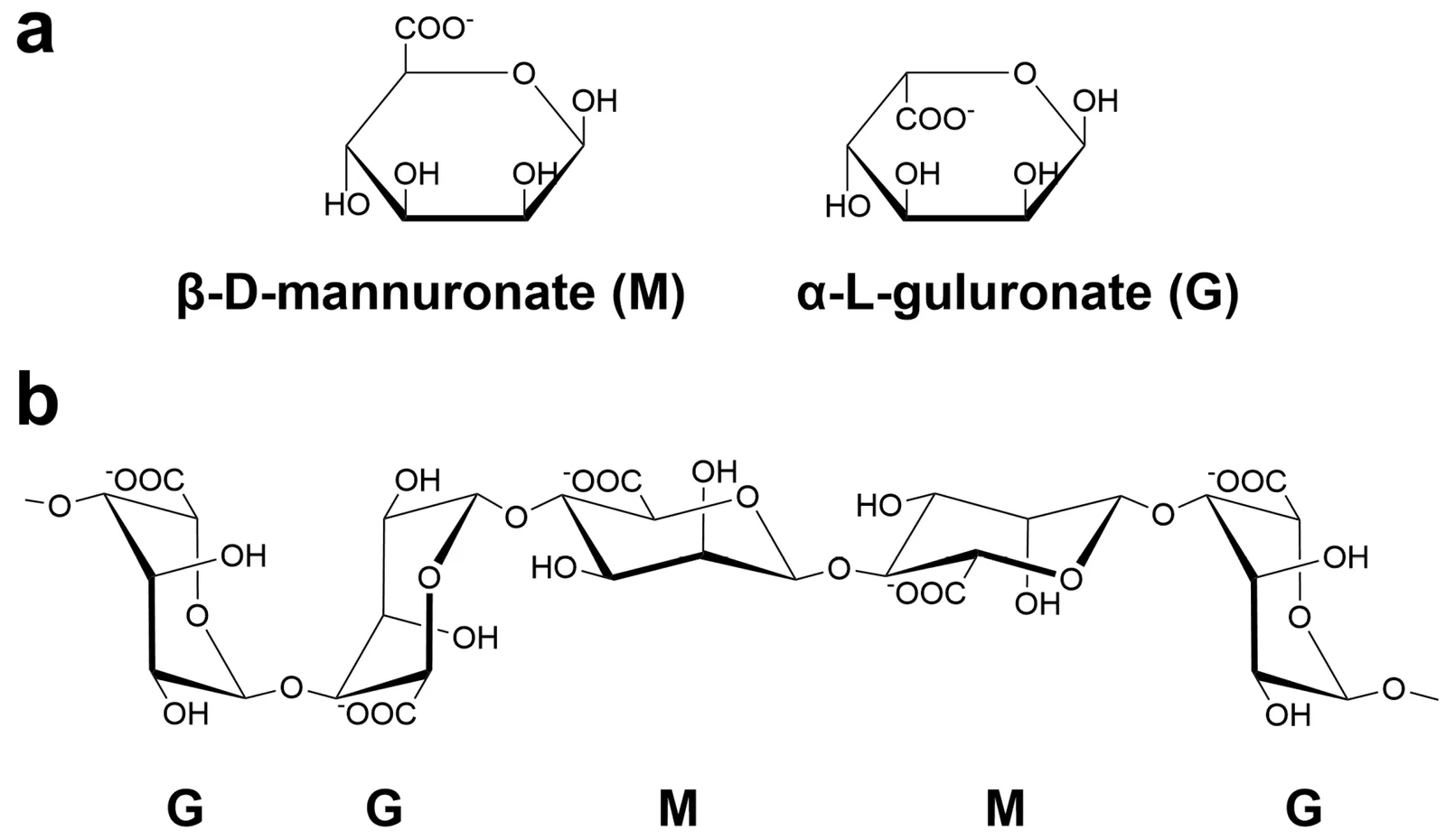
Structural characteristics of alginate: (**a**) chemical structures of β-D-mannuronate (M) and α-L-guluronate (G) monomers; (**b**) chain conformation showing polyM, polyG, and polyMG block arrangements.

Alginate is built from β-D-mannuronate (M) and α-L-guluronate (G) joined by 1,4-glycosidic linkages (Figure 2a,b). Rather than a simple random copolymer, the chain is organized in a highly aperiodic block pattern comprising three types of domains, namely consecutive polymannuronate blocks (polyM), polyguluronate blocks (polyG), and alternating M/G blocks (polyMG), whose lengths and proportions vary markedly with species, tissue, and even growth season [4,5]. Although the two uronate residues are C-5 epimers, their pyranose-ring conformations differ fundamentally: M residues adopt the ^4^C_1_ chair, whereas G residues adopt the ^1^C_4_ chair. This endows the two block types with entirely different chain geometries: polyG blocks form a rigid, extended zig-zag conformation, while polyM and polyMG blocks are comparatively flexible.

On the macroscopic scale, this conformational difference is embodied in the egg-box model proposed by Grant et al. [12] in 1973. The carboxyl and hydroxyl groups of G residues in adjacent polyG segments are arranged in layers to form cavities that cooperatively chelate divalent cations such as Ca^2+^, generating an intermolecularly cross-linked gel network in which the sequestered metal ions sit like eggs in an egg box. Egg-box cross-linking gives GG-rich alginate its high-strength gel properties; it is both the molecular basis of the mechanical integrity of brown-algal cell walls and the chemical principle underlying medical sodium-alginate gel materials [4]. Accordingly, the M/G ratio has become the core index governing the application performance of alginate: G-rich alginates form stiff, brittle gels suited to wound dressings and cell immobilization, whereas M-rich alginates form flexible gels that degrade faster and are suited to sustained drug release.

For alginate lyases, this block architecture means that their substrate is essentially a heterogeneous mixture presented in homopolymeric form: the enzymes must recognize not only the chemical identity of the uronate residues but also the sequence information of M and G. This requirement is the structural root of the broad substrate-specificity spectrum of the lyase family, which spans polyM-specific, polyG-specific, and bifunctional types, as detailed in Section 4.

### 2.2. Brown-Algal Origin and the Renewable Carbon-Source Attribute

In brown algae, alginate, cellulose, and fucoidan together form the cell-wall matrix network that gives the thallus flexibility and resistance to wave impact; alginate can account for 17–45% of the dry weight, depending on species and season. Giant brown macroalgae such as the kelps *Saccharina* and *Macrocystis* achieve annual biomass growth rates far exceeding those of terrestrial crops without requiring arable land or freshwater, and are widely regarded as third-generation biorefinery feedstocks [9,10].

The economics of brown-algal biorefining, however, have long been constrained by the efficiency of sugar-platform generation. The high molecular weight and viscosity of alginate render algal hydrolysates colloidal, severely impeding mass transfer and downstream fermentation, whereas chemical acid hydrolysis lacks block selectivity and produces by-products under strongly acidic conditions, raising downstream separation costs. Enzymatic depolymerization centered on alginate lyases is therefore regarded as the key enabling technology for unlocking the entire brown-algal biorefining process [13,14]. Notably, the composition of the enzymatic hydrolysate directly determines its downstream value, and this composition spans the DP distribution, the M/G composition, and the unsaturated termini: unsaturated AOS of DP 2–10 display pronounced activities in plant-growth promotion, antioxidation, and prebiotic applications [15,16], whereas the DEH monosaccharide released by exhaustive depolymerization enters microbial central metabolism and supplies carbon for fermentation platforms [9,10]. This link between product specificity and application value is a major motivation for enzymological and engineering research on alginate lyases.

### 2.3. Bacterial Origin and the Role as a Biofilm Virulence Factor

Unlike brown-algal alginate, bacterial alginate is synthesized as a polymannuronate backbone that undergoes post-synthetic epimerization and almost always carries O-acetyl groups (Section 2.4). The primary alginate-producing bacteria species are the soil diazotroph *Azotobacter vinelandii* and the opportunistic pathogen *P. aeruginosa* [17]. In *A. vinelandii*, extracellular alginate serves as a cyst-wall component protecting the cell under stress and, together with alginate lyases, participates in wall remodeling during cyst germination. One of the three lyases characterized by Gimmestad et al. [18] participates in this process, revealing a physiological role for lyases in the bacterial life cycle. The bacterium also secretes extracellular AlgE-family C-5 epimerases that convert polyM chains into products containing polyMG or polyG blocks. This system is not only a classic model for studying the block biosynthesis of alginate but also a unique tool for the industrial, enzymatic tailoring of alginates with defined M/G compositions [17].

In PA, by contrast, extracellular alginate is among its most important virulence factors. Alginate synthesis is carried out by a membrane-located complex encoded by the *alg* operon, in which AlgG combines C-5 epimerase activity with a chain-protective role adjacent to the polymerase, ensuring that nascent polyM chains are modified and exported before they can be degraded by the periplasmic lyase AlgL [19]. In the CF lung, environmental stresses such as hyperosmolarity, oxidative stress, and antibiotics continuously select for mutants that overproduce alginate, most commonly through inactivation of *mucA*. In these mutants, upregulation of the alginate-biosynthesis pathway drives the mucoid phenotype and secretion of large amounts of highly acetylated alginate [20]. In mucoid PA biofilms, alginate acts together with other extracellular polysaccharides such as Pel and Psl to establish a diffusion barrier that maintains sub-lethal local antibiotic concentrations while shielding bacteria from neutrophil oxidative burst and phagocytosis [3,19]. High-level acetylation contributes substantially to this barrier. Limoli et al. [21] delineated the multifaceted roles of EPS in biofilm structural integrity, antibiotic tolerance, and immune evasion, and Nivens et al. [22] further demonstrated in O-acetylation-deficient mutants that loss of acetylation markedly compromises microcolony structure and stability—directly establishing the biological significance of this modification.

The intracellular homeostasis of mucoid PA also depends on the lyase. Periplasmic AlgL degrades alginate that fails to be exported, preventing periplasmic accumulation that would otherwise trigger lethal stress. When the balance between synthesis and export is disrupted, however, AlgL itself—acting on its retained substrate—promotes cell death [23,24]. This dual role of AlgL as intracellular scavenger and lethal switch constitutes a striking physiological paradox and points to a promising antivirulence strategy: disrupting AlgL expression or activity to destabilize homeostasis in mucoid bacteria [25,26].

### 2.4. O-Acetylation and Its Impact on Enzymology and Medical Applications

O-Acetylation represents the most fundamental difference between bacterial and brown-algal alginate. M residues in PA alginate are acetylated at O-2 or O-3, whereas brown-algal alginate carries no acetylation at all [22]. Franklin et al. [27] identified the *algI*/*algJ*/*algF* gene cluster responsible for this modification and delineated the pathway by which acetyl groups are transferred from acetyl-CoA to the growing polymer.

O-acetylation profoundly affects enzymatic degradation. Most marine bacterial lyases naturally target non-acetylated alginate and show markedly reduced activity on acetylated substrates. At the molecular level, the specific residues governing acetyl group sensitivity remain unidentified due to the scarcity of enzyme complexes with acetylated alginate. Two mechanisms are likely involved. Acetyl groups at O-2 and O-3 may clash with side chains at the +1 and +2 subsites, particularly in enzymes with narrow binding clefts, and acetylation also masks hydroxyl groups, disrupting the hydrogen-bond network that anchors the substrate. These mechanisms align with the observation that PL6 and PL18 enzymes are more strongly affected than certain PL7 enzymes with wider and more open clefts [23,24,25,26]. Quantitatively, acetylation can reduce activity by up to tenfold in PL6 and PL18 enzymes, whereas some PL7 enzymes retain substantial activity, and higher degrees of substitution generally correlate with greater activity loss. Systematic dose–response data and position-specific analyses remain largely unavailable, although current evidence suggests that O-3 acetylation may interfere more directly with subsite recognition while O-2 acetylation may pose greater steric hindrance at the cleft entrance. Resolving these relationships will require standardized alginate substrate panels with controlled acetylation degrees and regiospecificity, combined with detailed kinetic analysis across multiple enzyme families. Beyond its effect on enzymatic degradation, acetylation also contributes substantially to the immunogenicity and biofilm-barrier function of alginate [22].

The difference in acetylation status creates a conspicuous substrate mismatch at the heart of current alginate lyase research. The overwhelming majority of enzymatic characterizations and protein-engineering efforts use commercial, non-acetylated sodium alginate as the model substrate, whereas the true target of antibiofilm applications is highly acetylated bacterial alginate. Judging the medical potential of an enzyme solely from activity data obtained on non-acetylated substrates may therefore severely overestimate its real-world efficacy. Commercial sodium alginate, used in most enzymological studies, has key limitations. Its M/G ratio and molecular weight vary across batches and are often poorly specified, and industrial extraction can alter native block structure. Most critically, commercial alginate entirely lacks O-acetylation, whereas bacterial alginate is heavily acetylated. Activity data from commercial substrates may therefore not reflect performance on native alginate, underscoring the need for standardized, well-characterized substrate panels. The AlyP1400 series screened by the Daboor group from marine bacteria has attracted attention precisely because these enzymes retain degrading activity against the EPS of mucoid PA biofilms under conditions mimicking CF sputum [28,29]. Future discovery and engineering of lyases specific for, or tolerant of, O-2 and O-3 acetylation will be the key direction for bridging the gap between industrial enzymology and clinical translation, as discussed further in Section 7.

In summary, alginate functions as both an industrial feedstock and a virulence barrier, drawing two research communities to the same enzyme family. Seaweed biorefining seeks industrial enzymes combining high activity, high stability, and controllable degradation products. Anti-infective research sets a higher bar for medical enzymes, which must retain activity in CF sputum and effectively degrade acetylated alginate while remaining compatible with existing antibiotic regimens. Both fields share a scientific foundation in dissecting polymer structure–enzyme recognition relationships, and their respective performance demands are increasingly addressed by the taxonomic, structural biology, and protein-engineering studies reviewed below.

## 3. Classification and Catalytic Mechanisms of Alginate Lyases

### 3.1. The CAZy Classification System and Overview of PL Families

Polysaccharide lyases (PLs) are enzymes that cleave glycosidic bonds through β-elimination. Unlike hydrolases, which use a water molecule as the nucleophile, PLs operate under basic conditions: a proton neutralizes the negative charge of the C-5 carboxylate, the leaving group at C-4 departs, and a C-4–C-5 double bond is formed. The product shows a characteristic absorbance at 235 nm, which conveniently enables activity quantification [5,6]. Lombard et al. [7,8] established in the CAZy database a hierarchical classification framework based on sequence similarity and structural fold, providing a unified taxonomic language for alginate lyases. As of 2025, the PL families known to contain alginate lyases include at least PL5, PL6, PL7, PL14, PL15, PL17, PL18, PL31, PL32, PL34, PL36, PL39, PL41, and PL44, and new families continue to emerge. For example, Zhou et al. [30] reported in 2024 the first member of a polyM-preferring, entirely new family, indicating that the sequence diversity of these enzymes is far from exhausted by the current classification. Garron and Cygler [31] classified the uronate-containing PL families mechanistically from a structural-chemistry perspective, noting that despite highly divergent fold topologies, the catalytic strategies display a striking coherence of chemical logic driven by convergent evolution.

**Figure 3:**
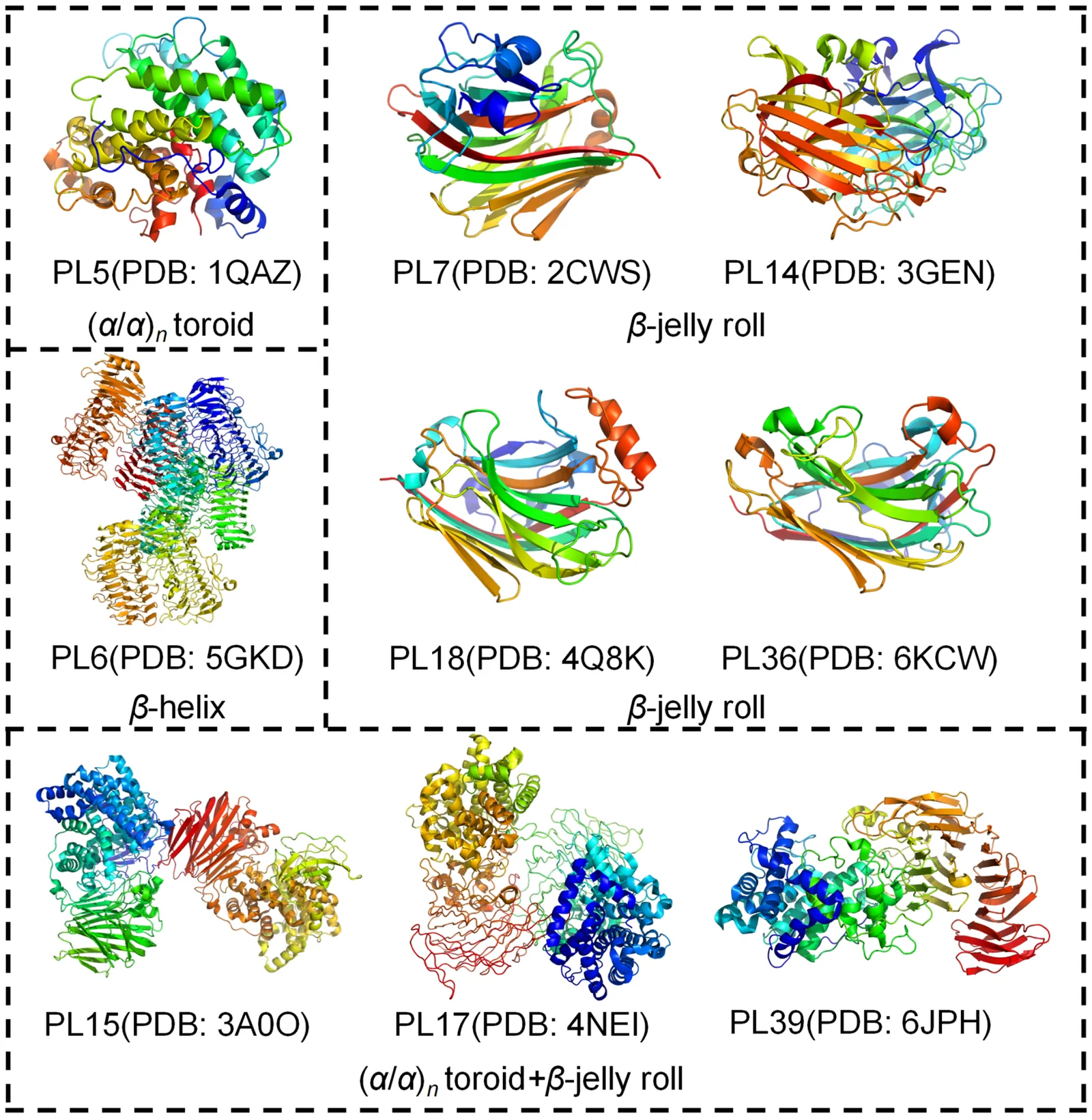
Three-dimensional structures of alginate lyases from different families.

The overall structures of alginate lyases fall into four classes (Figure 3): the β-jelly roll, adopted by enzymes of the PL7, PL14, PL18, and PL36 families and exemplified by A1-II′ of PL7 under PDB accession 2CWS [32,33]; the (α/α)n toroid, typical of PL5 enzymes and exemplified by A1-III under PDB accession 1QAZ [34]; the β-helix, typical of PL6 enzymes and exemplified by AlyGC under PDB accession 5GKD [35,36]; and a composite architecture combining an (α/α)n toroid with a β-jelly roll, typical of PL15, PL17, and PL39 enzymes and exemplified by Atu3025 of PL15 under PDB accession 3A0O [37,38]. These structural differences indicate that the catalytic function arose independently multiple times during evolution: despite different folding scaffolds, the chemical constraints imposed by the same substrate ultimately selected functionally convergent active-site configurations. For example, the PL7 enzyme ALY-1 from a *Corynebacterium* species and the PL5 enzyme A1-III from *Sphingomonas* sp. A1 adopt a β-jelly roll and an (α/α)n toroid, respectively; the two belong to different genera and share no discernible sequence or topological similarity, yet the spatial arrangement of their catalytic residues is nearly superimposable [39], a classic case of convergent evolution.

### 3.2. Diversity of β-Elimination Catalytic Mechanisms

Although active-site composition varies markedly across families, all alginate lyases share the same β-elimination catalytic chemistry. The reaction begins with neutralization of the C-5 carboxylate negative charge by active-site elements—Ca^2+^, positively charged Arg/Lys side chains, or Gln/Asn amide side chains. This renders the α-proton H-5 more acidic; a His, Lys, or Tyr side chain then abstracts H-5 as a base, forming a C-5 carbanion intermediate, followed by C-4 glycosidic bond cleavage and formation of the unsaturated C-4=C-5 double bond [31]. Rivas-Fernández et al. [40] further resolved the stereochemical rules of proton abstraction through structural and kinetic analyses across families. The +1 subsite residue determines the relative orientation of H-5 and the cleavable glycosidic bond, with G placing them on opposite sides of the ring and M on the same side. Accordingly, G-specific enzymes follow only a trans-elimination pathway, M-specific enzymes only cis-elimination, and bifunctional enzymes alternate between the two depending on substrate residue. While β-elimination chemistry is universal, active-site element combinations differ substantially among families.

Representative PL-family enzymes and their catalytic residues, cleavage mechanisms, and substrate preferences are listed in Table 1. Among structurally characterized representatives, the classic two-residue His/Tyr system is the most common, with His abstracting H-5 and Tyr donating the proton. In the PL7 enzyme A1-II′, the catalytic base is His191 and the catalytic acid Tyr284 [32,33]; in the PL15 enzyme Atu3025, they are His311 and Tyr365, respectively [37]; and in the PL39 enzyme Dp0100, they are His405 and Tyr239; the enzyme’s open-ended catalytic canyon endows it with an endolytic mode and a broad substrate spectrum spanning polyM and polyG [41]. When the +1 subsite residue is M, H-5 and the leaving group lie on the same side of the sugar ring, so a single Tyr residue can perform both steps of abstraction and proton donation in sequence; Tyr246 of the PL5 enzyme A1-III [34] and Tyr353 of the PL18 enzyme aly-SJ02 [42] are representative of such single-residue bifunctional catalysis. Beyond His-containing systems, several atypical combinations exist. The PL17 exolytic enzyme Alg17c uses Tyr450 as the base and Tyr258 as the acid [43]. The PL36 enzyme Aly36B employs a single Lys143 as both acid and base, which represents the most economical catalytic solution known [44]. The PL6 exolytic enzyme AlyGC uses Lys220 as the base and Arg241 as the acid, relying on Ca^2+^ both to neutralize the carboxylate charge and to stabilize the transition state, a metal-assisted strategy that is particularly common among marine-derived enzymes [35,36].

In addition, the PL14 enzyme vAL-1 from chlorella virus displays a rare pH-dependent dual mode of action [45]. At neutral pH, it cleaves its native substrate, the glucuronate polysaccharide of the chlorella cell wall, in endolytic fashion, whereas under alkaline conditions it switches to an exolytic mode and progressively releases small oligosaccharides; Lys197 and Ser219 are essential for catalysis under both conditions, yet the complete catalytic mechanism of this family remains to be elucidated. Overall, alginate lyases share a unified β-elimination mechanism yet employ diverse active-site configurations, a pattern that reflects convergent evolution and provides varied catalytic strategies for protein engineering.

## 4. Structural Basis of Substrate Recognition and Product Specificity

### 4.1. Classification of Substrate Specificity and Its Structural Determinants

The selectivity of alginate lyases for the three block types of polyM, polyG, and polyMG is the foremost feature determining product composition and, in turn, the direction of application. Based on substrate preference, the enzymes fall into three classes. The first are polyM-specific lyases, which mainly cleave consecutive MM linkages and yield oligosaccharides terminated predominantly in unsaturated M. The second are polyG-specific lyases, which correspondingly degrade GG blocks. The third are bifunctional lyases, which are active on both polyM and polyG and can also cleave the linkages of alternating MG regions, in principle enabling degradation of all block types of alginate [6,7]. Representative polyM-specific enzymes include the lyase from *Pseudomonas* sp. KS-408 [46] and a novel endolytic enzyme from another *Pseudomonas* species [47]. The foremost polyMG-specific enzyme is that from *Stenotrophomonas maltophilia* KJ-2 [48], which filled a long-standing gap in the enzyme toolbox for MG-block degradation. Bifunctional enzymes are exemplified by the PL18-family aly-SJ02 from *Pseudoalteromonas* sp. SM0524 and the enzyme from *Marinimicrobium* sp. H1 [49].

Substrate specificity is determined primarily by the shape and charge distribution of the active-site cleft and by side-chain orientations at substrate-binding subsites. Following Davies nomenclature, subsites are numbered −n to +n, with cleavage between −1 and +1. Structural studies of the PL7 enzyme A1-II′ [32,33] revealed that its active-cleft floor is curved to complement the substrate sugar rings, while aromatic residues lining the cleft provide binding energy through hydrophobic stacking and C–H···π interactions. The +1 and +2 subsites accommodate either G or M, while substrate carboxyl groups are recognized strictly by specific residues, which gives A1-II′ broad specificity toward polyM, polyG, and polyMG. MacDonald and Berger [50] identified, in the multifunctional PL enzyme Smlt1473, the switch residues that determine polyM versus polyG selectivity: replacing a single residue can markedly shift substrate preference, indicating that substrate specificity can be switched rapidly during evolution through a few mutations. Pandey et al. [51] further determined, at 1.5–2.0 Å resolution, the substrate-binding modes of this enzyme at pH 6 and pH 7, showing that the pH-dependent change in substrate selectivity arises from altered protonation states of residues at the +1 and +2 subsites, which fine-tune the binding geometry and thereby provide direct structural evidence for the design concept of steering product composition through environmental conditions.

Beyond block type preference, substrate M/G ratio and molecular weight also substantially influence measured enzyme activity and specificity. PolyG-preferring and polyM-preferring enzymes show higher activities on their respective block-rich substrates, while bifunctional enzymes vary with substrate composition [5,6,49]. Endolytic enzymes generally prefer high-molecular-weight substrates with more internal cleavage sites, whereas exolytic enzymes act most efficiently on short oligomers [52]. High-molecular-weight alginate also increases viscosity, which can reduce mass transfer and apparent activity at high substrate concentrations [13,14]. These parameters should therefore be fully specified for kinetic data to be comparable across studies.

### 4.2. Endolytic and Exolytic Modes and Their Topologies

A further division among PL families concerns mode of action. Endolytic enzymes cleave internal positions at random, rapidly reducing viscosity and producing oligosaccharides with a broad DP distribution. Exolytic enzymes successively remove mono- or disaccharides from the reducing or non-reducing end, fully depolymerizing oligosaccharides to metabolizable monosaccharides such as DEH. The active-site topologies of the two classes show a clear structure–function correspondence [37,43].

Endolytic enzymes have open canyons with both ends accessible, allowing long chains to thread through and undergo internal cleavage. In the PL39 enzyme Dp0100 catalytic domain complexed with an M5 pentasaccharide, Ji et al. [41] observed a ~50 Å canyon open at both ends. This open topology, distinct from the sealed pockets of exolytic enzymes, directly supports an endolytic mechanism. Exolytic enzymes have enclosed pockets accessible from one end only, restricting cleavage to terminal sugar units. The PL15 enzyme Atu3025 structure (Ochiai et al. [37]) reveals a blind-ended pocket accommodating only a trisaccharide and cleaving from the reducing end; the PL17 enzyme Alg17c (Park et al. [43]) is topologically similar. Despite low sequence similarity, catalytic-residue spatial layout is highly conserved, reflecting convergent evolution. The endolytic/exolytic classification is further supported by functional evidence. Endolytic enzymes rapidly reduce viscosity and substrate molecular weight through internal cleavage and produce a broad range of oligosaccharide DPs, whereas exolytic enzymes cause minimal viscosity change, act from chain termini, and release mainly monosaccharides and disaccharides. The exolytic *Shewanella* sp. Kz7 enzyme acts strictly on DP 2–4 oligosaccharides [53], whereas the endolytic Dp0100 processes long polysaccharide chains through its ~50 Å open canyon [41]. These functional profiles align with the observed open-canyon and sealed-pocket topologies. These two exolytic classes likely act cooperatively in marine bacterial degradation networks, whereby endolytic enzymes generate oligosaccharides that exolytic enzymes then break down to monosaccharides.

Beyond this dichotomy, several enzymes exhibit mode flexibility. The PL14 enzyme vAL-1 switches between endolytic and exolytic modes in a pH-dependent manner [45], and the *Stenotrophomonas* KJ-2 oligoalginate lyase operates bifunctionally [52], affording further control over product profiles.

### 4.3. Oligoalginate Lyases and Exolytic Degradation

Following oligosaccharide generation by endolytic enzymes, oligoalginate lyases further degrade these to the DEH monosaccharide, allowing bacteria to use alginate as the sole carbon source. Li et al. [53] first cloned and characterized a PL6-family oligoalginate lyase from *Shewanella* sp. Kz7, showing strict exolytic activity toward DP 2–4 oligosaccharides. Wang et al. [54] reported a similar enzyme from another *Shewanella* isolate, and Chu et al. [52] identified a KJ-2 oligoalginate lyase with both endolytic and exolytic activities, confirmed by site-directed mutagenesis. Together with endolytic enzymes, these oligoalginate lyases form a complete alginate-degradation network and provide key resources for integrated fermentation platforms converting brown algae to DEH [9].

### 4.4. Bifunctional Enzymes and Control of the Product Profile

In industrial applications, control over the DP distribution of enzymatic products directly determines AOS product quality [15,16]. Bifunctional endolytic enzymes degrade both polyM and polyG blocks and can therefore generate more uniform oligosaccharide profiles. Yet even under identical reaction conditions, different enzymes produce markedly different DP distributions. Cheng et al. [55] reported a novel bifunctional endolytic enzyme whose degradation patterns shift with substrate concentration and reaction time. This finding suggests that reaction engineering offers an effective lever for product control by tuning enzyme dosage, substrate loading, and reaction duration. Peng et al. [56] subsequently described a versatile bifunctional enzyme capable of generating both monosaccharides and oligosaccharides, opening a new route toward full utilization of all alginate components. In work on module truncation, Han et al. [57] systematically truncated Aly5 from *Flammeovirga* sp. MY04. Removing the carbohydrate-binding module (CBM) markedly shifted the product DP distribution toward shorter oligosaccharides. The authors concluded that the CBM anchors the enzyme to high-molecular-weight substrates and extends its residence time at endolytic sites, which indirectly shapes the final product profile. Yan et al. [49] applied site-directed mutagenesis to the bifunctional enzyme from *Marinimicrobium* sp. H1 and showed that catalytic performance can be enhanced without compromising bifunctionality.

### 4.5. Cold-Adapted and Eukaryotic Alginate Lyases

Lyases from extreme ecological niches have attracted growing interest in recent years. Chen et al. [58] identified a cold-adapted, salt-activated PL7 lyase from the deep-sea bacterium *Pseudoalteromonas* sp. SM0524. Its optimal temperature is only 25 °C, and its activity increases substantially in the presence of NaCl, reflecting the enzymological adaptations of marine halophilic and psychrophilic microorganisms. In 2025, He et al. [59] showed that a conserved motif in the PL7 family directly governs cold adaptability, offering useful enzyme candidates for low-temperature AOS production. Among eukaryotes, Inoue et al. [60] reported the first eukaryotic PL7-family alginate lyase from the red alga *Pyropia yezoensis*, establishing that the family also exists in eukaryotic algae. This finding points to possible horizontal gene transfer of alginate lyases between prokaryotes and eukaryotes. It also suggests that the plant kingdom itself possesses the enzymatic machinery to degrade alginate, likely acquired from epiphytic or parasitic brown algae.

### 4.6. Model Microorganisms and the Dissection of Degradation Networks

*Zobellia galactanivorans* serves as the model bacterium for brown algal degradation. In 2012, Thomas et al. [61] conducted a systematic genome-wide CAZyome analysis of this species and identified multiple PL7 lyases arranged in clusters designated alginolytic operons. Their work revealed a division-of-labor organizational principle in the alginate degradation network. Endolytic enzymes, oligoalginate lyases, and transporters are co-expressed at the operon level, creating an efficient carbon flow from extracellular polymers into central metabolism. Genomic studies of model microorganisms like this one provide a reference framework for mining new enzymes from metagenomic data. A comparative analysis of the three lyases from *Pseudomonas fluorescens* HZJ216 [62] uncovered within-strain heterogeneity. The three enzymes differ in substrate preference, optimal pH, and product distribution, and these differences likely reflect distinct physiological roles such as nutrient acquisition and alginate turnover. The alginate-degrading properties of *Agarivorans* sp. B2Z047 [63], together with subsequent validation of its ability to degrade fresh and dried macroalgae directly [64], make this strain a candidate for one-step bacteria-algae biorefining.

In summary, the lyase families span a broad spectrum of substrate specificities, modes of action, and ecological origins, providing a diverse enzyme repertoire from which application developers can select candidates suited to particular process requirements.

## 5. Protein Engineering Strategies for Alginate Lyases

Protein engineering of alginate lyases has focused primarily on improving their performance in applied settings, including thermostability, catalytic efficiency, substrate and product specificity, and enzyme immobilization. Guided by bioinformatic and structural analyses of existing enzymes, researchers have deployed site-directed mutagenesis, computational design, domain recombination, and immobilization to endow the engineered enzymes with the desired properties, thereby better meeting the demands of industrial and medical applications. This section reviews progress in alginate lyase engineering from five angles: the need for engineering and the limitations of native enzymes, rational design based on disulfide bonds and hydrogen-bond networks, strategies for breaking the activity–stability trade-off, computational and artificial-intelligence-assisted enzyme design, and enzyme immobilization with process integration. Table 2 summarizes the engineering strategies and outcomes of representative case studies.

### 5.1. The Need for Engineering and the Performance Limitations of Native Enzymes

Although nature offers an abundant reservoir of alginate lyases, their direct deployment in industrial or medical settings is frequently constrained by three bottlenecks. The first is insufficient thermostability: most marine-derived enzymes have optimal temperatures of 35–50 °C, whereas industrial hydrolysis reactions often must run at 45 °C or above for extended periods, and thermal inactivation keeps the cost of enzyme replenishment stubbornly high [13,14]. The second is the trade-off between catalytic efficiency and stability, in which gains in stability are usually purchased at the price of conformational flexibility and, with it, catalytic efficiency; breaking this trade-off has been a long-standing theme of protein engineering [69]. The third is limited substrate adaptability: native enzymes show low activity toward acetylated alginate or high-molecular-weight substrates, and the DP distribution of their products is difficult to control precisely [13,65]. To address these bottlenecks, researchers have developed a diversified toolkit of engineering strategies, ranging from rational design through directed evolution to computational assistance, and the families targeted for engineering have expanded from PL7 and PL18 to broader families such as PL6 and PL39 [14,65].

### 5.2. Rational Design Based on Disulfide Bonds and Hydrogen-Bond Networks

Rational thermostabilization centers on introducing extra disulfide bonds and reinforcing hydrogen-bond networks and hydrophobic packing. In cAlyM, a PL7-family enzyme from *Microbulbifer* sp. Q7, Yang et al. [65] used molecular dynamics (MD) simulations to identify loop regions that become excessively flexible at elevated temperatures, and rationally designed the D102C/A300C double mutation to install an intramolecular disulfide bond. The half-life of the mutant at 45 °C was extended from 1.9 h to 4.15 h and its Tm rose by 1.7 °C; moreover, the optimized loop conformation raised its specific activity and catalytic efficiency to 1.8- and 1.5-fold those of the wild type, respectively. This study provided the first systematic demonstration of an engineering paradigm in which MD-guided disulfide design achieves a coordinated improvement in stability and activity.

The hydrogen-bond network strategy has since been developed further. In 2025, Jiang et al. [66] identified key hydrogen-bond nodes in Alyw203, a PL7-family enzyme, through computational analysis and rebuilt the network with multiple point mutations, markedly improving both thermostability and catalytic efficiency while keeping the product profile unchanged. Zhang et al. [67] introduced several stabilizing mutations into FlAlyA by computer-assisted rational design while preserving high expression levels, yielding a candidate enzyme for industrial-scale AOS production. Guo et al. [68] rationally thermostabilized a PL6-family enzyme, further demonstrating that the strategy is transferable across families.

### 5.3. Strategies for Breaking the Activity–Stability Trade-Off

A long-standing challenge in protein engineering lies in the inherent trade-off between stability and activity. Stabilizing mutations often restrict conformational flexibility, dampening the dynamic motions that underpin catalytic turnover and resulting in diminished activity. Huang et al. [69] addressed this dilemma through a sequential two-step engineering approach applied to the PL7-family enzyme AlyG2. In the first step, the T91S substitution widened the substrate tunnel entrance and relieved product diffusion constraints, yielding a 1.91-fold increase in specific activity. The second step introduced the S72Y mutation onto this already improved variant, which enhanced thermotolerance at 42 °C without compromising the activity gains. This sequential strategy outperforms the conventional paradigm where stabilization is typically achieved at the expense of catalytic performance. Further analyses of hydrogen-bond dynamics and essential motion subspaces elucidated the underlying thermostabilization mechanism, providing a mechanistic basis for the broader applicability of this approach.

In a complementary study, Su et al. [70] targeted both a flexible loop and the substrate entrance of a PL18-family enzyme. The resulting triple mutant E226K/I211T/R294V exhibited a 4.8-fold improvement in catalytic efficiency and a substantial extension of half-life at 45 °C from 89 min to 560 min. Molecular dynamics simulations attributed these gains to enhanced loop flexibility and a widened substrate access channel. Collectively, these studies demonstrate that alginate lyases can be engineered through either sequential optimization, where activity enhancement precedes stabilization, or simultaneous co-engineering of both properties, offering flexible paradigms for future enzyme improvement efforts. Despite these advances, important limitations remain. Both the sequential two-step approach and simultaneous co-engineering have been demonstrated in only a handful of enzymes, and their generalizability to PL families with distinct fold topologies remains uncertain. Most engineering studies assess activity and stability under standard laboratory conditions, whereas real applications involve combined stresses including elevated temperature, high ionic strength, and proteolytic degradation that are seldom evaluated together. Whether enzymes optimized under single-stress conditions maintain performance under multi-stress environments remains unresolved. Product profile is also rarely considered in current engineering studies, and whether activity and stability gains can be achieved without altering product DP distribution remains unknown.

### 5.4. Computational and Artificial-Intelligence-Assisted Enzyme Design

Whereas traditional rational design depends on a researcher’s intuitive understanding of a particular enzyme, computational design is driving a paradigm shift. In 2025, Ma et al. [71] reported a rational computational design strategy for VxAly7B-CM that used FoldX and Rosetta ddG to predict the energetic effects of mutations, combined with MD-based flexibility assessment, and thereby screened out mutants with simultaneously improved catalytic activity and thermostability and validated the feasibility of a computational screening workflow for alginate lyases. In a complementary vein, He et al. [59] combined computational and experimental analyses of conserved motifs in PL7-family enzymes to reveal the decisive role of motif engineering in cold adaptation.

More broadly, the protein-engineering toolkit is being reshaped by AlphaFold 2/3 structure prediction, HotSpot Wizard 3.0 automated mutation-hotspot identification, and machine-learning stability prediction via ProteinMPNN and ESM-1v scoring [65]. While these tools remain early-stage in alginate lyase research, experience with cellulases and amylases suggests that generative-AI de novo design and large-scale virtual mutant-library screening will become major growth areas within 3–5 years. Several promising directions emerge from this shift. Researchers may move beyond the boundaries of natural sequence space, engineer catalytic domains that recognize non-natural substrates including O-acetylated forms, and build multifunctional fusion enzymes that integrate endolytic and exolytic catalytic domains within a single tandem architecture.

### 5.5. Enzyme Immobilization and Process Integration

Beyond engineering at the molecular level, immobilization and process integration are equally decisive for industrial deployment. In 2024, Kaur et al. [72] reported the immobilization of an alginate lyase on surface-modified carriers, improving thermostability and reusability while maintaining the product profile and thereby providing a viable route toward industrial AOS production in enzyme–membrane coupled reactors. The modular-truncation work of Han et al. [57] and the computational design of Zhang et al. [67] represent parallel efforts to optimize product profiles and expression levels from the perspectives of molecular structure and modularity, respectively.

In summary, alginate lyase engineering has evolved from the early empirical mode of single-point mutation followed by activity testing into a structure- and computation-guided discipline that integrates multiple engineering strategies. The families under engineering have expanded from PL7 to PL6, PL18, and beyond, and the engineering objectives have grown from thermostability alone to the coordinated multi-objective optimization of activity, stability, and product profile. The challenge for the next stage is to couple computational and AI tools with high-throughput screening so as to deliver precisely tailored enzymes for specific applications, such as acetylated substrates, low-temperature processes, and antibiofilm uses.

## 6. Applications of Alginate Lyases

### 6.1. Targeted Preparation of Alginate Oligosaccharides and Structure–Activity Relationships

Alginate oligosaccharides are unsaturated or saturated alginate fragments with degrees of polymerization of 2 to 10 and are the most important direct products of alginate lyases. In a systematic review published in 2023, Li et al. [15] summarized the preparation strategies, structural features, and structure–activity relationships of AOS. The preparation strategies span acid hydrolysis, enzymatic degradation, and physical degradation, while the structural features include the M/G composition, the degree of polymerization, and whether the termini are saturated. Unsaturated AOS display pronounced antioxidant, antitumor, prebiotic, and plant-growth-promoting activities, and these activities are closely tied to both the DP and the M/G ratio, with unsaturated M-derived oligosaccharides of DP 3–5 generally being the most active. Among these activities, antioxidant and plant-growth-promoting effects are supported by both in vitro assays and in vivo plant models, whereas antitumor and prebiotic effects rely mainly on in vitro evidence with limited in vivo validation. Structural determinants differ by activity type, as antioxidant activity correlates most strongly with the unsaturated terminal double bond and DP 3 to 5, whereas prebiotic effects depend more on the M/G ratio and resistance to gastrointestinal digestion. The molecular mechanisms underlying these activities, including specific cellular targets and signaling pathways, remain largely hypothetical and require further validation. From the structure–activity perspective, Lu et al. [16] further pointed out that the double bond at the unsaturated terminus is the chemical basis of most of the observed bioactivities. This structural signature corresponds precisely to the products of lyase-catalyzed β-elimination, which underscores the irreplaceable role of enzymatic routes in preparing bioactive oligosaccharides.

Targeted preparation means selecting the appropriate enzyme combination and reaction conditions according to the DP and composition of the desired product. For example, to obtain DP 5–10 polyM-type oligosaccharides, one should choose a polyM-specific endolyase and terminate the reaction at low conversion; to achieve complete degradation to the DEH monosaccharide, a combination of endolyases and oligosaccharide exolyases is required [9,53,54]. Bifunctional and cold-adapted enzymes expand the palette of process options by preserving activity at low temperature [58].

### 6.2. Seaweed Biorefining and Integrated Enzymatic Hydrolysis–Fermentation

At the broader level of biomass utilization, alginate lyases constitute the pivotal link in the route from brown seaweed to DEH and onward to fermentation products. In their milestone study of 2012, Wargacki et al. [9] integrated the 36 kb alginate-utilization gene cluster of *Vibrio splendidus* into *Escherichia coli* and paired it with a secreted lyase system, uniting degradation, uptake, and metabolism in a single host that fermented brown seaweed directly into ethanol at a volume fraction of 4.7%, approximately 80% of the theoretical yield. This work provided the first demonstration that all components of brown seaweed can be utilized within one engineered strain, marking the decisive step of seaweed biorefining from proof of concept to engineering reality.

Enquist-Newman et al. [10] subsequently engineered a *Saccharomyces cerevisiae* platform by integrating the fungal DEHU transporter Ac_DHT1, bacterial alginate-metabolism genes, and a deregulated mannitol pathway, achieving an ethanol titer of 4.6% (*v*/*v*) at 83% of the theoretical yield and proving that yeast can exploit all three major sugars of brown seaweed, namely mannuronic acid, guluronic acid, and mannitol. Sasaki et al. [73] then simplified the process with a co-culture of two engineered yeasts, one dedicated to degradation and the other to fermentation; although the titer was somewhat lower, the gain in process flexibility was substantial. The shared lesson of these studies is that the heterologous expression efficiency, secretion level, and substrate adaptability of the lyase are the key variables limiting overall process economics, so enzyme engineering and chassis-cell engineering must advance in concert.

An alternative route to biomass utilization is the direct use of whole cells to degrade fresh or dried seaweed, bypassing enzyme isolation altogether. In 2024, Gong et al. [64] reported that *Agarivorans albus* B2Z047 degrades both fresh and dried seaweed directly, releasing reducing sugars without any pretreatment, and thereby offered a candidate strain for one-step biorefining [63,64]. Such whole-cell catalysts are attractive because they eliminate the cost of enzyme purification, but their degradation rates and product controllability still require optimization.

While these studies establish the technical feasibility of brown-algal biorefining, process engineering and economic considerations remain underexplored. Industrial-scale alginate lyase production relies mainly on recombinant expression in bacterial or yeast chassis, and enzyme cost, determined by expression level, secretion efficiency, and purification, remains a major economic factor, although detailed cost data are rarely reported publicly. Enzymatic depolymerization offers milder conditions, higher selectivity, and fewer inhibitory by-products than acid hydrolysis, but requires higher enzyme input and typically achieves lower volumetric productivity. Enzyme-membrane coupled reactors can partially offset these drawbacks through continuous enzyme recycling and product removal [72]. Systematic techno-economic and life cycle assessments remain scarce and will be essential to define the cost thresholds at which enzymatic routes become competitive, especially for high-value products such as bioactive AOS.

### 6.3. Antibiofilm Medical Applications and Preclinical Studies

Whereas industrial applications pursue complete degradation, antibiofilm applications aim instead at selectively dismantling the EPS barrier and restoring antibiotic susceptibility. The review by Koo et al. [74] systematically laid out the challenges and strategic prospects of biofilm therapy, listing matrix-degrading enzymes among the representative anti-virulence and barrier-disrupting strategies; alginate lyases are prominent members of this enzyme class.

The starting point for understanding this application is AlgL, the periplasmic lyase encoded by *Pseudomonas aeruginosa* itself, which scavenges alginate molecules that fail to be exported to the exterior and thereby preserves periplasmic homeostasis. Jain et al. [23] showed that AlgL is essential for alginate transport in mucoid PA: its loss causes alginate to accumulate in the periplasm and kills the cell. Albrecht et al. [25] went further and demonstrated that AlgL activity is itself a prerequisite for alginate biosynthesis. Bakkevig et al. [26] established the scavenger function of AlgL in P. fluorescens, and the early work of Monday et al. [24] likewise supported a cooperative role for AlgL and AlgX in alginate synthesis. That a single enzyme acts as both scavenger and lethal switch is an apparently paradoxical physiology, yet it offers a unique entry point for anti-virulence drug design: interfering with AlgL expression or activity can destabilize the homeostasis of mucoid bacteria and thereby enable the enzyme to serve synergistically as an antibiotic adjuvant.

The concept of combining enzymes with antibiotics was established by Alkawash et al. [11] in 2006, in the first systematic validation of the enhanced killing of mucoid PA biofilms by an exogenous lyase paired with antibiotics. In a continuous-flow biofilm model mimicking CF sputum, gentamicin alone was virtually ineffective, whereas lyase pretreatment markedly reduced the viable cell count within the biofilm, and the combination cleared the biofilm synergistically. In 2016, Germoni et al. [75] further showed that the lyase-mediated reversal of gentamicin resistance is biofilm-specific, with no significant effect on planktonic cells, implying that the enzyme acts by dismantling the EPS barrier rather than by killing bacteria directly. Tavafi et al. [76] cloned a novel lyase gene from PA TAG 48 itself and verified its biofilm-clearing activity against PA, indicating that even autologously derived enzymes possess antibiofilm activity.

The series of studies on the marine enzyme AlyP1400 represents the landmark breakthrough in this direction. AlyP1400, isolated by the Daboor group from a marine *Pseudoalteromonas* sp. [28], became a key research model because it retains the capacity to degrade the EPS of mucoid PA biofilms under conditions mimicking CF sputum. Their follow-up work [29] demonstrated that AlyP1400 combined with tobramycin or ciprofloxacin substantially reduces the biofilm biomass of the CF clinical isolate CF27 and enhances bacterial killing. Particularly telling is the observation that heat-inactivated enzyme loses the synergistic effect: catalytic activity is thus a necessary condition for synergistic clearance, which directly confirms a mechanism in which enzymatic degradation of the barrier restores antibiotic penetration.

A ternary system combining enzyme, antibiotic, and nanomaterial marks the further evolution of antibiofilm therapy. In 2020, Wan et al. [77] constructed silver-nanoparticle mesoporous organosilica particles of approximately 39 nm co-loaded with an alginate lyase and ceftazidime, in which the lyase serves as a targeting ligand that binds the alginate matrix, the silver nanoparticle acts as both an antibacterial agent and carrier, and ceftazidime provides the antibiotic payload. In a murine model of pulmonary PAO1 infection, this system achieved targeted release at pH 6.4 along with pronounced biofilm clearance and no evident toxicity. The ternary construct exemplifies the progression of antibiofilm therapy from a single enzyme adjuvant toward multi-component, cooperatively acting smart delivery systems, and it opens new avenues for clinical translation.

The potential risks of enzyme–antibiotic combination therapy were revealed by Daboor et al. [78] in 2021. Flow-cytometric analysis showed that biofilm cells dispersed upon simultaneous release of enzyme and tobramycin upregulated virulence- and resistance-related genes, including algR, bdlA, lasB, mexF, mexY, and ndvB, and that roughly 60% of the dispersed cells became tolerant to a subsequent antibiotic challenge. This timing effect likely reflects the narrow window between cell dispersal and antibiotic exposure, during which dispersed cells can upregulate resistance mechanisms and survive subsequent treatment. Crucially, potentiation occurred only with simultaneous rather than sequential administration, underscoring the importance of dosing-timing design in the clinic. Beyond dosing timing, co-administration with anti-quorum sensing agents and nanoparticle-based co-delivery systems that synchronize enzyme and antibiotic release [77] offer additional routes to ensure that dispersed cells are immediately exposed to bactericidal concentrations. The phenotypic heterogeneity and dissemination risk of dispersed cells thus sound a warning for the in vivo safety design of enzyme-assisted antimicrobial strategies.

Although OligoG CF-5/20, a low-DP alginate oligosaccharide preparation composed of small G-block oligosaccharides with DP 2–10, is not itself a lyase, it is a product of lyase degradation and likewise displays antibiofilm adjuvant activity. Hengzhuang et al. [79] demonstrated in a murine pulmonary infection model that OligoG CF-5/20 disrupts the structure of mucoid PA biofilms and potentiates antibiotic clearance. This represents a second route by which lyase-derived products synergize with antibiotics: the degradation product itself acts as an antibiofilm adjuvant, so that substrate hydrolysis and product-mediated antimicrobial action operate as a cascade. Key parameters of these enzyme–antibiotic combination studies, including antibiotic class, biofilm model, and main finding, are summarized in Table 3.

Safety considerations span three areas. Degradation products such as AOS are generally biocompatible [15], but toxicological data for high-dose or prolonged exposure remain limited, and the metabolic fate of DEH in the host is unclear. Exogenous enzymes of marine bacterial origin such as AlyP1400 carry immunogenic risk with repeated aerosol administration, as heterologous proteins may induce neutralizing antibodies or pulmonary inflammation [28]. Aerosolized enzymes also face rapid mucociliary clearance and proteolytic degradation in CF sputum, which limit lung retention, whereas nanoparticle encapsulation [77] can protect the enzyme and prolong residence at the biofilm site. Clinical safety data for intact enzymes remain scarce, with most evidence coming from oligosaccharides rather than enzyme products. Released oligosaccharides can also exert DP-dependent immunomodulatory effects [16]. Most critically, dispersed cells show upregulated virulence and antibiotic resistance genes [78], raising concerns of transiently increased pathogenicity and systemic dissemination, which underscores the need for optimized enzyme dosing and timed antibiotic co-administration. Early clinical data with OligoG suggest acceptable safety [79], although larger studies are needed.

In summary, antibiofilm applications have advanced from the 2006 proof of concept by Alkawash et al. [11] to the 2020 in vivo validation of the ternary nanosystem by Wan et al. [77], with marked gains in both research depth and strategic sophistication. Viewed from the standpoint of clinical translation, however, substrate adaptability remains the central gap. The lyases with the best-established efficacy are mostly of marine bacterial origin, and their natural substrate is non-acetylated alginate, whereas the real substrate awaiting them in CF sputum is heavily acetylated bacterial alginate. AlyP1400 earned its status as a star molecule precisely because it overcame this gap, yet it remains an isolated case. Future work must systematically evaluate additional enzymes against acetylated substrates and, through protein engineering, create enzymes with high activity toward them. In parallel, safety considerations including degradation product biocompatibility, enzyme immunogenicity, and dispersed-cell risks must be systematically addressed as the field moves toward clinical translation.

## 7. Challenges and Future Perspectives

### 7.1. Adaptation to Acetylated Substrates and Engineering Strategies

As discussed in Chapters 2 and 6, O-acetylation remains the key gap between fundamental research and clinical application of alginate lyases. Most current enzymological characterizations and protein-engineering efforts use commercial sodium alginate as the model substrate, and this substrate carries no acetyl groups; as a result, kinetic parameters such as Km, kcat, and kcat/Km may deviate substantially from the performance that would be obtained in authentic medical settings. Sporadic studies have shown that the effect of acetyl groups is enzyme-dependent: certain PL7 enzymes are barely affected, whereas the activity of some PL6 and PL18 enzymes drops by as much as an order of magnitude. Systematic comparative data, however, remain extremely scarce.

Future work should advance simultaneously on the substrate, mechanism, and engineering fronts. On the substrate front, a panel of standardized bacterial alginates should be established covering acetylation at O-2, at O-3, and at both positions, and degrees of substitution ranging from 0 to 0.8; such a library would serve as a universal substrate set for activity assessment and replace the single sodium alginate model substrate [22,27]. On the mechanism front, crystal structures of enzyme–acetylated substrate complexes should be solved and combined with molecular dynamics simulations to clarify how acetyl groups contribute to, or detract from, subsite binding energy and where their steric clashes originate, thereby providing a structural foundation for rational design. On the engineering front, directed evolution and computational design can screen for acetylation-tolerant pocket mutants. Mechanism-guided redesign offers clear principles, as bulky residues at the +1 and +2 subsites can be replaced with smaller residues such as Ala, Val, or Leu to create steric room, and hydrophobic residues can be introduced to favor interactions with the acetyl methyl group, while catalytic residues and the key hydrogen-bond network must be preserved to maintain activity. Molecular dynamics simulations and pocket volume analysis can prioritize mutations before experimental testing. Consequently, enzyme performance data obtained on non-acetylated substrates cannot be directly extrapolated to medical settings, and the field currently lacks a validated framework for predicting in vivo efficacy from in vitro kinetic parameters.

### 7.2. Artificial-Intelligence-Driven Enzyme Design Paradigms

The next wave of transformation in protein engineering is being driven by artificial intelligence. Although applications of AlphaFold 3, ProteinMPNN, and the ESM series in the alginate lyase field are still in their infancy, several paths forward can already be foreseen. AlphaFold 3 enables high-accuracy structure prediction of families lacking crystal structures, such as PL31, PL41, and PL44, thereby narrowing the gap between structurally characterized and sequence-only families [40]. HotSpot Wizard 3.0 can identify mutation hotspots balancing conservation and flexibility, combining FoldX-based stability prediction with enzyme-function score-based activity prediction to achieve multi-objective virtual screening. De novo design of miniature catalytic domains tailored for non-natural substrate preferences, including specificity toward acetylated substrates or MG blocks, would enable researchers to move beyond the constraints of natural sequence space [14,69,71]. Multifunctional fusion enzymes represent another promising direction, including constructs that integrate endolytic and exolytic catalytic domains in a single tandem architecture for one-step alginate degradation, or lyase antimicrobial peptide fusions that couple degradation with direct bacterial killing.

### 7.3. Multi-Objective Optimization of Activity, Stability, and Product Profiles

Classical engineering focuses on a single objective such as thermostability or activity, whereas practical applications require coordinated multi-objective optimization. Industrial AOS production requires enzymes with high activity, high stability, and a controllable product profile, whereas medical applications demand enzymes that retain activity under CF sputum conditions characterized by low oxygen, neutral pH, high ionic strength, and neutrophil proteases while remaining compatible with antibiotic regimens. The two-step strategy reported by Huang et al. [69] has demonstrated the feasibility of co-optimizing activity and stability, yet systematic engineering of the product profile dimension remains largely unexplored. Going forward, a multi-objective evaluation matrix should be established that spans activity, stability, product DP distribution, and adaptability to acetylated substrates, and high-throughput product-analysis methods such as HPLC-MS/MS or capillary electrophoresis should be incorporated into the screening workflow, so that product-profile information becomes available already at the mutant-library screening stage [15,16].

### 7.4. Safety and Dosing Regimens of Antibiofilm Strategies

The warning raised by the upregulation of virulence and resistance genes in dispersed cells [78] indicates that the in vivo safety of enzyme-assisted antimicrobial therapy demands rigorous evaluation. Dosing schedule optimization requires clarifying whether sequential administration, in which enzyme pretreatment precedes the antibiotic, or simultaneous administration performs better across different infection models, to avoid treatment failure caused by dispersed-cell tolerance. Virulence gene monitoring should track the in vivo expression dynamics of genes such as algR and lasB in dispersed cells to assess long-term dissemination risk. Delivery system optimization should extend the pH-triggered release and spatially coordinated co-release of enzyme and antibiotic embodied in the ternary nanosystem [77] to additional enzyme–antibiotic combinations. Compatibility with existing CF therapy regimens also requires preclinical validation, particularly the pairing of nebulized lyase with the mucolytic dornase alfa and formulation stability alongside inhaled antibiotics such as tobramycin, aztreonam, and fosfomycin.

### 7.5. Integrated Enzyme Engineering and Process Design

On the industrial side, enzyme engineering must advance in parallel with process integration. Enzyme membrane-coupled reactors, which combine continuous enzymatic hydrolysis with ultrafiltration membranes for simultaneous product separation and enzyme recycling, avert product inhibition, and the immobilization work of Kaur et al. [72] provides a foundation for this direction. Coordinating whole-cell catalysis with free-enzyme processes is another key consideration, as the direct bacterial seaweed degradation of Gong et al. [64] suits front-end pretreatment, whereas back-end high-precision tailored hydrolysis requires free enzymes, and deploying the two in tandem balances efficiency with controllability. AI-assisted process optimization can further reduce costs, as machine-learning models that predict optimal enzyme combinations and reaction conditions from kinetic parameters and substrate properties can curtail experimental trial and error.

### 7.6. Mining New Families and New Ecological Niches

The number of families in the CAZy database continues to grow [8,30], a sign that the sequence and ecological diversity of alginate lyases is far from exhausted. Four directions of exploration merit particular attention. The first is enzymes from the gut microbiota: for example, the structure of BcAlyPL6, resolved by Wang et al. [80] from the human gut bacterium *Bacteroides clarus*, revealed the enzymological basis by which gut bacteria exploit dietary alginate, a direction closely tied to research on microbiome–host interactions. The second is phage-derived enzymes: alginate lyases encoded in phage genomes might be deployed as lysogenic lysis strategies against mucoid PA, complementing lyase-based antibiofilm therapy. The third is enzymes from extreme environments: thermophilic enzymes from deep-sea hydrothermal vents, psychrophilic enzymes from polar regions, and halophilic enzymes from hypersaline habitats each possess distinct properties and can supply customized candidates for specific processes or medical conditions [58,59]. The fourth is eukaryotic enzymes: the PL7 enzyme from the red alga *Pyropia* [60] demonstrates that the family also exists in eukaryotes, hinting that alginate lyases may play ecological roles in plant–alga interactions.

## 8. Conclusions

As the only biocatalysts capable of specifically depolymerizing alginate, a polymer drawn from two distinct origins, alginate lyases occupy a central position in both brown-seaweed biorefining and antibiofilm therapy. Under the theme of one enzyme connecting two substrate origins and serving two application directions, this review systematically surveys progress in the alginate lyase field from 2005 to 2025. In terms of substrate chemistry, the block architecture and O-acetylation of alginate together define the chemical basis of lyase substrate specificity. At the catalytic mechanism level, more than twelve PL families recognized in the CAZy system exhibit both convergent evolution and fold diversity, with active-site configurations ranging from His/Tyr and Tyr/Tyr pairs to bifunctional Lys, metal-assisted catalysis, and pH-dependent switches. For substrate recognition, the open canyons of endolytic enzymes and the sealed pockets of exolytic enzymes, together with subsite residue switching and the product controllability of bifunctional enzymes, underpin product specificity. Protein engineering has advanced from rational disulfide design through the two-step optimization strategy and hydrogen-bond network reconstruction to AI-assisted design. On the application side, industrial uses include targeted AOS preparation, seaweed biorefining platforms, and whole-cell direct degradation, whereas medical applications encompass AlgL physiology, combined enzyme and antibiotic therapy, ternary nanosystems, and safety considerations raised by dispersed cells.

This review advances a central argument that adaptability to O-acetylated substrates represents the critical gap between industrial enzymology and medical translation. Current research exhibits a pronounced substrate mismatch, as enzymological characterization and engineering rely almost exclusively on non-acetylated sodium alginate, whereas the relevant medical target is heavily acetylated bacterial alginate. Closing this gap will require the development of standardized acetylated substrate libraries, systematic evaluation of existing enzymes against these substrates, and protein engineering to generate enzymes with high activity toward acetylated alginate. Furthermore, the upregulation of virulence and resistance genes in dispersed cells warrants careful consideration in the safety design of enzyme-assisted antimicrobial strategies for in vivo applications.

Future prospects should focus on five directions: systematic breakthroughs in acetylated-substrate adaptability; AI-driven de novo design and virtual screening; coordinated multi-objective optimization of activity, stability, and product profiles; safety and dosing-schedule optimization for antibiofilm strategies; and full-chain integration from enzyme to process. These directions collectively converge on a unifying vision in which alginate lyases transition from model subjects of enzymological inquiry into genuine biotechnology tools capable of delivering on both industrial and medical promise, underpinned by systematic understanding of the interactions among polymer, enzyme, microbe, and host.

## Figures and Tables

**Table 1:** Overview of the characteristics of alginate lyase PL families.

PL Family	Enzyme	Catalytic Residues	Mode	Substrate Preference	Main Product (DP)	Catalytic Efficiency
PL5	A1-III [34]	Tyr	Endo	polyM	2-3	n.r.
PL6	AlyGC [35,36]	Lys, Arg	Exo	polyG	1 (unsaturated monouronate)	n.r.
PL7	A1-II′ [32,33]	His, Tyr	Endo	polyG & polyM	2-3	Km 1.51 µg/mL; kcat 46.3 min^−1^
PL14	vAL-1 [45]	Lys, Ser	Endo/exo	polyG & polyM	6 at pH 7; 2 at pH 10	n.r.
PL15	Atu3025 [37,38]	His, Tyr	Exo	Oligomeric termini	1 (Δ, then DEH)	Km 9.4 µM; 15.3–18.0 µmol/min/mg
PL17	Alg17c [43]	Tyr	Exo	Oligomeric termini	1 (Δ)	n.r.
PL18	aly-SJ02 [42]	Tyr	Endo	polyG & polyM	2–3 from polyM; 3–4 from polyG	Km lower for polyG than for polyM
PL36	Aly36B [44]	Lys	Endo	polyM	2	Km 0.46 mg/mL on alginate, 0.30 mg/mL on polyM
PL39	Dp0100 [41]	His, Tyr	Endo	polyG & polyM	2-4	Lowest Km within the truncation series

n.r., not reported in the primary reference; Δ, 4-deoxy-L-erythro-hex-4-enopyranuronate; DEH, 4-deoxy-L-erythro-5-hexoseulose uronate, the ketose formed by hydration of Δ.

**Table 2:** Protein engineering strategies and quantitative performance improvements of alginate lyases.

Enzyme	Strategy	Result
cAlyM	Disulfide design	Half-life at 45 °C: 1.9→4.15 h; Tm +1.7 °C; specific activity 1.8-fold; kcat/km 1.5-fold [65]
Alyw203	H-bond network redesign	Thermostability and catalytic efficiency improved [66]
FlAlyA	Computer-aided design	Thermostability improved, high expression retained [67]
AlyRm6A	Rational stabilization	Thermostability improved [68]
AlyG2	Two-step strategy	Specific activity 1.91-fold; thermotolerance enhanced at 42 °C [69]
PL18 lyase	Loop & entrance engineering	kcat/km 4.8-fold; half-life at 45 °C: 89→560 min [70]
VxAly7B-CM	Computational screening	Activity and thermostability improved [71]
A1603	Immobilization	Stability and reusability improved [72]

**Table 3:** Comparison of alginate lyase–antibiotic combination studies.

Study	Enzyme	Antibiotic	Biofilm Model	Main Finding
Alkawash et al. [11]	Exogenous lyase	Gentamicin	Continuous-flow biofilm	Synergistic clearance; gentamicin alone nearly ineffective
Germoni et al. [75]	Exogenous lyase	Gentamicin	Standard biofilm	Biofilm-specific resistance reversal; no effect on planktonic cells
Daboor group [28,29]	AlyP1400	Tobramycin/ciprofloxacin	Biofilm (CF sputum-mimicking)	Reduced biofilm biomass; enhanced bacterial killing
Wan et al. [77]	Lyase	Ceftazidime	Murine lung infection (in vivo)	Targeted clearance; no evident toxicity

## Data Availability

No new data were created or analyzed in this study. Data sharing is not applicable to this article.

## Abbreviations

The following abbreviations are used in this manuscript:

EPS	Extracellular polymeric substance
PA	*Pseudomonas aeruginosa*
CF	Cystic fibrosis
DP	Degree of polymerization
AOS	Alginate oligosaccharides
DEH	4-deoxy-L-erythro-5-hexoseulose uronate
polyM	Polymannuronate block
polyG	Polyguluronate block
PL	Polysaccharide lyase
CBM	Carbohydrate-binding module
MD	Molecular dynamics
